# Executive anosognosia in progressive supranuclear palsy versus Parkinson’s disease

**DOI:** 10.3389/fneur.2026.1744979

**Published:** 2026-02-02

**Authors:** L. Ye, L. Seidler, D. Chemodanow, G. Respondek, C. Niesmann, I. Wilkens, M. Klietz, G. U. Höglinger, B. Kopp

**Affiliations:** 1 Department of Neurology, Hannover Medical School, Hannover, Germany; 2 Department of Neurology, Ludwig-Maximilians-University of Munich, Munich, Germany; 3 German Center for Neurodegenerative Diseases (DZNE), Munich, Germany; 4 Munich Cluster for Systems Neurology (SyNergy), Munich, Germany

**Keywords:** anosognosia, executive function, Parkinson’s disease, patient-reported outcome measures, progressive supranuclear palsy

## Abstract

**Background:**

Executive function deficits are common among patients with Parkinson’s disease (PD) and progressive supranuclear palsy (PSP). Executive function refers to higher-order cognitive processes thought to involve fronto-striatal circuits. Some patients with executive deficits may be unable to recognize or report them, a condition we refer to as executive anosognosia.

**Objective:**

To conduct a comparative analysis of executive anosognosia in patients diagnosed with PSP and PD.

**Methods:**

We compared an objective neuropsychological assessment (ONA) of composite executive function (ONA-CEF), which includes semantic and phonemic verbal fluency, as well as two sub-scores from the Wisconsin Card Sorting Test, with patient- and informant-reported rating scales. We used the Dysexecutive Questionnaire Revised (DEX-R) to evaluate near-transfer executive complaints and the Aachen Activity and Participation Index: Cognition and Participation (AAPI-CP) composite to evaluate far-transfer cognitive and social difficulties. Discrepancy indices were calculated for patients and informants (ONA-CEF minus DEX-R and ONA-CEF minus AAPI-CP).

**Results:**

PSP patients had significantly larger negative discrepancies than PD patients, indicating stronger executive anosognosia. Although informant reports reduced these discrepancies, significant underreporting persisted in PSP informants. Correlational analyses revealed that patient-reported DEX-R difficulties were strongly correlated with depressive symptoms (*r* ≈ 0.65) but not with objective executive performance (*r* ≈ 0.00).

**Conclusion:**

Executive anosognosia is a marker of PSP, highlighting the need for objective neuropsychological assessments in clinical trials. PSP patients’ reports of executive dysfunction are more associated with mood than actual impairment, which challenges the validity of patient-reported outcomes in PSP and related neurological diseases.

## Introduction

For several decades, clinicians and researchers have struggled with a persistent discrepancy between patients’ reports of cognitive and functional impairments and their performance on neuropsychological tests for various neurological disorders (1–3). This discrepancy is particularly evident in executive functions (4), such as working memory, planning, inhibitory control, and cognitive flexibility. Impairments in these functions can lead to severe difficulties with everyday problem-solving and social interactions. Self-report questionnaires aim to bridge the gap by probing patients’ perceptions of their everyday failures in executive control (5). However, these questionnaires depend on intact self-awareness and may be distorted by mood disturbances, personality traits, and metacognitive biases (6, 7). Consequently, they may underrepresent or overrepresent objective executive dysfunction, which could limit their trustworthiness as stand-alone cognitive outcome measures.

At the heart of this objective-subjective discrepancy lies anosognosia, or impaired awareness of one’s own deficits (2). Studies using neuroimaging and lesion analysis have linked anosognosia to dysfunction in frontal networks, particularly in the orbitofrontal, medial prefrontal, and anterior cingulate cortices (8–10). These regions are responsible for performance monitoring, self-evaluation, and metacognition (10, 11). For instance, up to 45% of patients with Parkinson’s disease (PD) without dementia underreport executive difficulties, even when objective testing reveals clear deficits in executive functions (7). Depressive symptoms and fatigue can also skew self-report data, sometimes inflating complaints regardless of actual cognitive ability (6, 12, 13). When completed by informants such as spouses or caregivers, these scales often reveal more severe dysexecutive problems than those acknowledged by the patients themselves. These findings underscore the need to validate patient- and informant-reported information to more accurately understand the role of self-reporting in this field.

Progressive supranuclear palsy (PSP) may be an even more striking example of anosognosia in executive dysfunction. PSP is characterized by early and pronounced frontal lobe atrophy (14). Consequently, patients exhibit significant impairments on executive testing (15–18). However, their awareness of these deficits is unclear. Theoretical models suggest that PSP’s more extensive frontal lobe pathology should produce greater objective-subjective discrepancies, or more severe executive anosognosia, than PD. Nevertheless, no study has systematically paired executive testing with multiple self-report instruments at varying “transfer distances” (from “near” questionnaires that address difficulties in executive control to “far” questionnaires that address broader cognitive and social domains) in these populations. Addressing this gap is critical for developing accurate assessment protocols that account for both objective test performance and patients’ insight into their daily executive functioning challenges.

The aim of this study is to investigate anosognosia for executive function in patients with PSP in comparison to patients with PD, all without dementia. We hypothesize that PSP patients will exhibit significantly larger negative discrepancy scores than PD patients, reflecting more pronounced executive anosognosia. Although informant reports are expected to reduce these discrepancies, we do not expect them to eliminate them entirely. Since negative emotion can distort self-reporting, we were also interested in understanding how mood-related biases may influence subjective reporting.

## Methods

### Patients and informants

This cross-sectional, observational study recruited 65 patients (38 with PSP and 27 with PD) from the Department of Neurology at Hannover Medical School, Germany, between 2020 and 2023.

Inclusion criteria included a probable PSP (19) or PD (20) diagnosis by a movement disorder specialist, proficiency in German, and a caregiver’s willingness to provide input (referred to as an “informant”). An informant was defined as someone who spends at least 5 h per week in close contact with the patient and has observed disease progression and daily abilities for at least 2 years. Patients with cognitive symptoms that prevented them from completing study procedures were excluded. Specifically, patients with a Montreal Cognitive Assessment (MoCA) score below 17, indicating severe cognitive impairment, were excluded.

Patients and caregivers received no financial or other compensation. The total assessment duration was approximately 2 h, with breaks allowed between sections.

Patients and informants provided written informed consent. The study was approved by the local ethics committee at Hannover Medical School (No. 9029_BO_S_2020, issued May 14, 2020) and was conducted in accordance with the Declaration of Helsinki.

### Neuropsychological measures

Our Objective Neuropsychological Assessment (ONA) uses four z-scores—two from verbal fluency tasks (semantic and phonemic) and two from the Modified Wisconsin Card Sorting Test (categories correct and perseverative errors)—to generate a composite executive functioning index (see below for details). These z-scores are adjusted for age, gender, and education to ensure that each component corrects for individual demographic characteristics. The combined measure is more reliable and valid than any single measure of executive functioning because it better captures the cognitive skills people use to handle everyday executive challenges.

The Dysexecutive Questionnaire–Revised (DEX-R) is a 37-item instrument available in patient-reported and informant-rated formats (21). It is designed to evaluate the everyday cognitive, emotional, motivational, and behavioral challenges associated with executive dysfunction. Based on Stuss and Alexander’s model of frontal lobe function (22), the DEX-R was validated in a study of individuals with acquired brain injuries, as well as their informants. Rasch analysis supported the construct validity of four theoretically derived subscales, with consistent results across patient and informant ratings. Subsequent research has confirmed that the DEX-R effectively discriminates between levels of executive dysfunction in clinical populations and detects age-related changes in nonclinical samples (23). However, mood disorders such as depression and anxiety can influence DEX-R scores (23). Overall, the DEX-R represents a near-transfer level of subjective reporting. Discrepancies between executive ONA and patient-reported DEX-R scores may indicate near-transfer executive anosognosia associated with neurological disease. Additionally, discrepancies may be observed between patient-reported and informant-reported DEX-R scores.

We used the Aachen Activity and Participation Index (AAPI) to assess broad real-world functional limitations (24). The AAPI measures activities of daily living across four subscales: Mobility (AAPI-M; 10 items), Activities (AAPI-A; 15 items), Cognition (AAPI-C; 14 items), and Participation (AAPI-P; 15 items). Although these four domains target motor, instrumental, cognitive, and social difficulties, our exploratory factor analysis supported a two-factor structure (details not reported here): physical functioning (AAPI-MA, combining mobility and activities items) and cognitive-social engagement (AAPI-CP, combining cognition and participation items). In our study, we focused on AAPI-CP (combined cognition-participation scores from 29 items) reported by patients and informants to capture perceived limitations in cognitive and social functioning. Overall, the AAPI-CP represents a far-transfer level of subjective reporting. Discrepancies between executive ONA and patient-reported AAPI-CP scores may indicate far-transfer executive anosognosia associated with neurological disease. Additionally, discrepancies may be observed between patient-reported and informant-reported AAPI-CP scores.

This study integrates an ONA based on a psychometrically optimized composite measure of executive functioning with subjective measures of near (DEX-R) and far (AAPI-CP) transfer from the perspectives of patients and informants within well-characterized samples of patients with PSP and PD. We calculated the following discrepancy indices: executive ONA minus DEX-R and executive ONA minus AAPI-CP, where larger negative discrepancies indicate greater executive anosognosia.

### Objective neuropsychological assessment

The examiner performed the neuropsychological assessments following training by an experienced clinical neuropsychologist. The Consortium to Establish a Registry for Alzheimer’s Disease (CERAD) Neuropsychology Assessment Battery (NAB) is a comprehensive tool designed to evaluate cognitive functions relevant to Alzheimer’s disease and related dementias. In German speaking countries, an extended CERAD NAB Plus is publicly available (25), which encompasses 13 key scores derived from the core CERAD NAB plus three added subtests, as detailed in Supplement A.

The Modified Wisconsin Card Sorting Test (M-WCST) is a widely used, standardized neuropsychological tool designed to evaluate executive functions such as cognitive flexibility and abstract reasoning, as detailed in Supplement B (26). The assessment captures key indices of executive function, including the number of categories correctly sorted (i.e., sets of six consecutive correct responses), the number of perseverative errors (i.e., repeated incorrect categorization strategies after receiving negative feedback), and a combined score called the “executive function composite (EFC).”

### Questionnaires

The DEX-R is a measure of executive dysfunction in daily life, especially following brain injury (21). Based on the original DEX (27), the DEX-R incorporates Stuss and Alexander’s model of frontal lobe function to evaluate the cognitive, behavioral, motivational, and emotional aspects of executive dysfunction (22). We used both the patient-reported (DEX-R-r) and the informant-reported (DEX-R-i) versions, which have identical items and scoring. Items (e.g., “I have difficulty planning ahead,” “I act without thinking”) are rated on a five-point scale (0 = never, 4 = very often), with total scores ranging from 0 to 148. Higher scores indicate greater dysfunction. For discrepancy analyses (see below), DEX-R scores were reverse-coded to align with neuropsychological scoring conventions.

The Aachen Activity and Participation Index (AAPI) evaluates functional limitations in daily life (24). Based on its two-factor structure, we used the AAPI-CP (Cognition and Participation) to assess cognitive-social participation and the AAPI-MA (Mobility and Activity) to assess mobility-related functioning. Both were administered as patient reports (AAPI-CP-r and AAPI-MA-r) and informant reports (AAPI-CP-i and AAPI-MA-i), with identical items covering domains such as managing finances and organizing social activities. There are 29 items for the AAPI-CP and 25 items for the AAPI-MA. Items are rated from 0 (“could not do it at all”) to 4 (“no problem at all”), yielding total scores of 0–116 for the AAPI-CP and 0–100 for the AAPI-MA. Lower scores indicate greater limitations, while higher scores reflect better functioning.

The Beck Depression Inventory–Fast Screen (BDI-FS) is a seven-item, self-report measure that assesses the severity of depressive symptoms (28). It focuses on psychological and cognitive aspects while excluding somatic symptoms. Items are rated on a four-point scale (total score: 0–21), with the following categories: minimal (0–3), mild (4–6), moderate (7–9), and severe (10–21). The BDI-FS shows strong convergent validity with other depression measures (29).

### Data analysis

The Objective Neuropsychological Assessment (ONA) Composite Executive Function (CEF) index is based on combining four z-standardized measures: semantic and phonemic verbal fluency (Extended CERAD NAB Plus) and categories achieved, as well as perseverative errors (M-WCST). This composite provides a reliable, multidimensional index of executive functioning (30).

All measures were z-transformed using the combined PD and PSP sample. The z-transformation yielded z(ONA-CEF), z(DEX-R-r/i), z(AAPI-CP-r/i), and z(BDI-FS), all with a mean of 0 and a standard deviation of 1.

For z(ONA-CEF), lower scores indicate poorer executive function. Since higher DEX-R scores indicate more severe symptoms, z′(DEX-R) was reverse-coded.


$$z^{'}(\mathrm{DEX}-R)=-z(\mathrm{DEX}-R).$$

Thus, lower z′(DEX-R) scores reflect greater symptom severity, aligning with z(ONA-CEF). No reverse coding was done for z(AAPI-CP) or z(BDI-FS) because higher scores already indicate greater severity.

To assess executive anosognosia, we calculated the near-transfer discrepancy as follows:


$$\text{Near}-\text{transfer discrepancy}=z(\mathrm{ONA}-\mathrm{CEF})-z^{'}(\mathrm{DEX}-R).$$

Negative scores indicate overestimation of executive abilities (executive anosognosia) and positive scores indicate underestimation (heightened awareness).

Far transfer of executive anosognosia was calculated as:


Far−transfer discrepancy=z(ONA−CEF)−z(AAPI−CP).

Negative values indicate anosognosia, meaning objective executive deficits exceed perceived cognitive-social impairment.

Discrepancies between self- and informant reports were also calculated:


DEX−Rdiscrepancy=z(DEX−R−r)−z(DEX−R−i).

AAPI−CPdiscrepancy=z(AAPI−CP−r)−z(AAPI−CP−i).

For the DEX-R, negative values suggest patient underreporting, and positive values suggest overreporting. For the AAPI-CP, the interpretation is reversed: positive values suggest underreporting and negative values suggest overreporting by patients relative to informants.

Together, these metrics quantify executive anosognosia by capturing discrepancies between objective performance and patient or informant perspectives.

Statistical analyses were conducted using JASP (v0.18.3)^1^ and R Studio (v2024.09.1 + 394). Comparisons of dependent correlations were conducted using Psychometrica,^2^ applying a method equivalent to Steiger’s *z*-test (31). This tool determines whether one correlation is significantly stronger than another within the same sample.

### Data sharing

The data that support the findings of this study are available from the corresponding author upon reasonable request.

## Results

### Clinical measures

Patients with PSP (*n* = 38; 36 PSP-RS and 2 PSP-PGF phenotype) and PD (*n* = 27) were assessed using clinical measures (Table 1). PSP patients showed greater overall severity (CGI-S, *p* < 0.01), shorter disease duration (*p* < 0.001), and more advanced morbidity (PSP stage, *p* < 0.05) than PD patients. Activities of daily living were more impaired in PSP (Schwab & England, *p* < 0.01). Motor scores (MDS-UPDRS III) and demographics did not differ significantly.

**Table 1:** Demographic and clinical characteristics of the progressive supranuclear palsy (PSP) and Parkinson’s disease (PD) groups.

Measures	PSP (*n* = 38)	PD (*n* = 27)	*p*
Age (years)	68.9 ± 8.8	69.9 ± 10.3	0.657
Gender (male) (%)	51.3	59.3	0.724
Duration of the disease (years)	3.7 ± 2.4	8.7 ± 6.3	<0.001***
CGI	4.0 ± 1.1	3.1 ± 1.1	<0.01**
Hoehn and Yahr	3.2 ± 0.6	2.8 ± 0.9	0.071
PSP stage	2.8 ± 0.8	2.0 ± 1.1	<0.05*
MDS-UPDRS III	30.8 ± 16.8	26.0 ± 11.9	0.250
PSPRS total score	28.5 ± 13.4	20.1 ± 9.3	<0.05*
SEADL (%)	64.0 ± 21.0	80.9 ± 10.0	<0.01**

Data are presented as mean ± SD. CGI: clinical global impression-severity scale; MDS-UPDRS III: movement disorder society-unified Parkinson’s disease rating scale part III; PSPRS: progressive supranuclear palsy rating scale; SEADL: Schwab and England activities of daily living. *p < 0.05, **p < 0.01, ***p < 0.001; analyzed with *R*.

### Exploring the neuropsychological measures of interest

For the neuropsychological data shown in Table 2, a MANOVA (Pillai’s trace) revealed a significant group effect (PSP versus PD) on CERAD-Plus variables (*F*(13, 35) = 3.12, *p* < 0.01). This effect was driven by lower semantic and phonemic fluency in PSP (both *p* < 0.001). There were no significant group effects for naming, memory, or visual tasks, except for more word list intrusions in PD (*p* < 0.05). The ONA-CEF score, based on four key variables, also revealed a significant group effect (*F*(4, 44) = 6.80, *p* < 0.001), which was again driven by differences in fluency. However, M-WCST measures did not differ significantly by group.

**Table 2:** Objective neuropsychological assessment of cognition in progressive supranuclear palsy (PSP) and Parkinson’s disease (PD) patients in terms of standardized *z* values.

Measures		PSP	PD
MoCA (required for inclusion decisions)		25.1 ± 2.8	24.3 ± 3.5
CERAD-Plus	**Semantic fluency**	**−2.1 ± 1.2**	**−0.7 ± 1.0**
	Abbreviated Boston Naming Test	0.0 ± 1.4	0.7 ± 0.6
	Wordlist learning	−1.1 ± 1.2	−0.6 ± 1.3
	Wordlist recall	−1.0 ± 1.0	−0.4 ± 1.3
	Wordlist intrusions	−0.5 ± 1.2	−1.1 ± 1.2
	Wordlist savings	−0.7 ± 1.4	−0.4 ± 1.5
	Wordlist discriminability	−0.7 ± 1.2	−0.9 ± 1.3
	Figure copying	−1.5 ± 1.5	−0.7 ± 1.6
	Figure recall	−1.1 ± 1.4	−0.7 ± 1.7
	Figure savings	−0.2 ± 1.4	−0.4 ± 1.5
	Trail Making Test A	−1.7 ± 1.3	−0.9 ± 1.4
	Trail Making Test B	−1.7 ± 1.2	−1.0 ± 1.3
	**Phonemic fluency**	**−1.2 ± 1.3**	**0.1 ± 1.0**
M-WCST	EFC (composite score)	−1.3 ± 1.3	−0.8 ± 1.1
	**Number of categories achieved**	**−1.4 ± 1.3**	**−1.1 ± 1.0**
	**Number of preservative errors**	**−1.0 ± 1.4**	**−0.5 ± 1.0**

Data are presented as *M* ± SD. Standardized *z*-values have a mean of 0 and a standard deviation of 1 in the reference population. Negative *z*-values indicate below-average performance, and the absolute value indicates the degree of deviation from the population mean. MoCA: Montreal cognitive assessment; CERAD: consortium to establish a registry for Alzheimer’s disease plus (CERAD-Plus): extended CERAD neuropsychological assessment battery; M-WCST: modified Wisconsin card sorting test. The four variables highlighted in bold were used to calculate the objective neuropsychological assessment-composite executive function (ONA-CEF) score.

**Figure 1:**
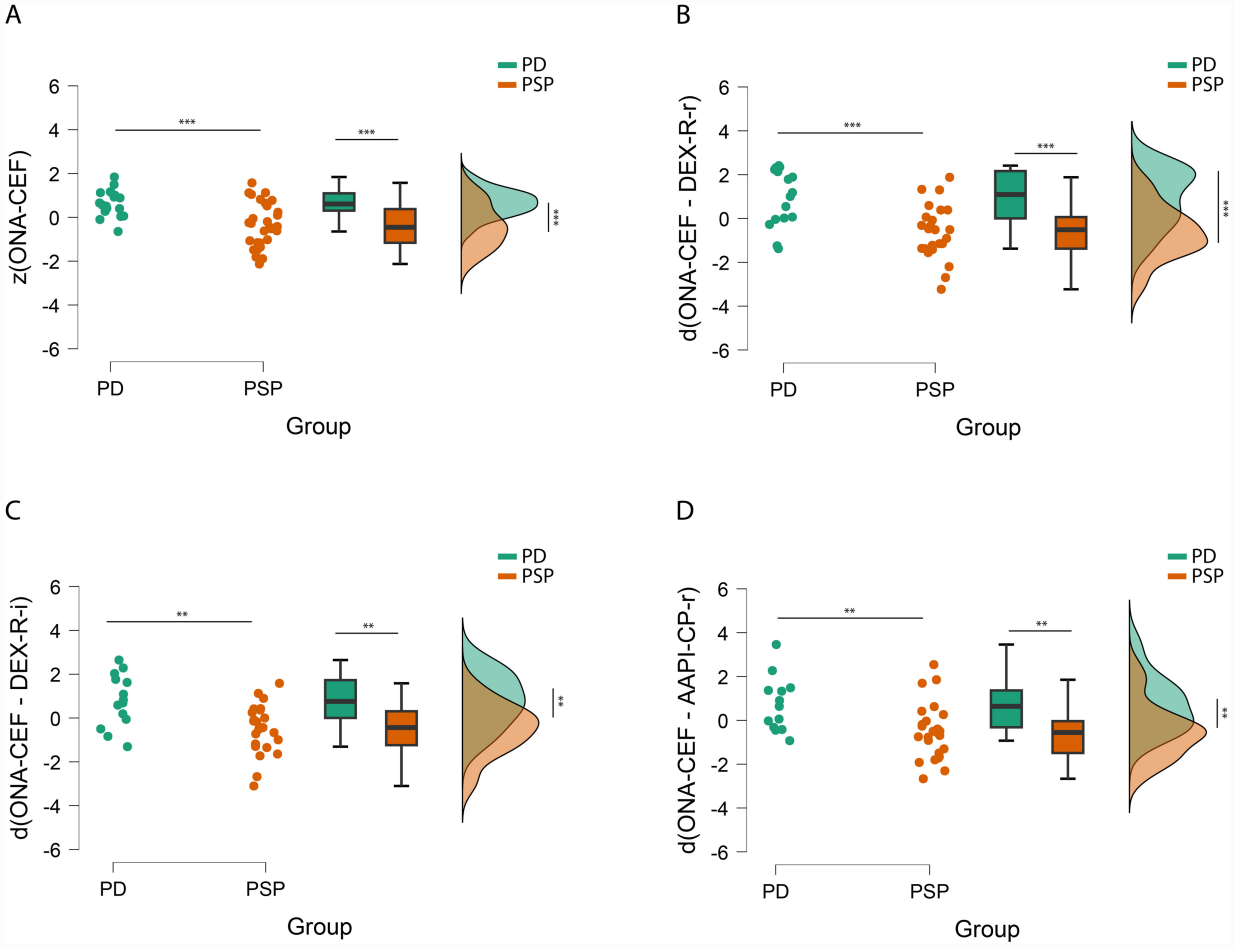
Scatter, box, and raincloud plots, separately for PD and PSP patients. **(A)** Standardized composite executive functioning scores. **(B)** Standardized discrepancies between objective executive function and patient-reported executive difficulties. **(C)** Standardized discrepancies between objective executive function and informant-reported executive difficulties. **(D)** Standardized discrepancies between objective executive function and patient-reported cognitive and participative difficulties. Green dots represent individual data points for the PD group; orange dots for the PSP group. *p* < 0.01, *** *p* < 0.001.

ANOVA revealed a significant group difference in composite executive functioning (ONA-CEF), *F*(1, 47) = 15.84, *p* < 0.001, *η*^2^*ₚ* = 0.252. PD patients (*M* = 0.652) outperformed PSP patients (*M* = −0.379), indicating poorer executive function in PSP patients (Figure 1A). For patient-reported executive dysfunction (DEX-R-r), ANOVA revealed a trend of higher reported problems in PD (*M* = 0.283) than in PSP (*M* = −0.217), with a *p*-value of 0.071 and an effect size of 0.062. Informant-reported executive dysfunction (z(DEX-R-i)) did not differ between groups, *F*(1, 46) = 0.001, *p* = 0.976. There were no significant group differences for patient- or informant-reported cognitive-social functioning (z(AAPI-CP-r), *F*(1, 47) = 0.36, *p* = 0.553; z(AAPI-CP-i), *F*(1, 42) = 0.67, *p* = 0.418). PSP patients showed significantly poorer objective executive function, but subjective reports (both patient- and informant-reported) were similar across groups. PD patients tended to report more executive difficulties than PSP patients, but this did not extend to functional participation.

### Exploring standardized differences as indicators of executive anosognosia

#### Near-transfer discrepancy (ONA-CEF minus DEX-R-r)

ANOVA revealed a significant group difference (Figure 1B) in the discrepancy between objective executive functioning and patient-reported dysfunction (*F*(1, 39) = 15.43, *p* < 0.001, *η*^2^*ₚ* = 0.284). Patients with PD showed a positive discrepancy (*M* = 0.938), indicating overreporting of problems. In contrast, patients with PSP showed a negative discrepancy (*M* = −0.639), indicating underreporting relative to impaired performance, which is consistent with executive anosognosia in PSP.

#### Near-transfer discrepancy (ONA-CEF minus DEX-R-i)

A significant group difference (Figure 1C) also emerged for the discrepancy between objective performance and informant-reported dysfunction, *F*(1, 35) = 10.67, *p* < 0.01, *η*^2^*ₚ* = 0.234. Informants of PD patients overreported problems (*M* = 0.788), while PSP informants underreported them (*M* = −0.506), suggesting reduced awareness of deficits in caregivers of patients with PSP.

#### Near-transfer discrepancy (DEX-R-r minus DEX-R-i)

The discrepancy between patient- and informant-reported dysfunction was not significant, *F*(1, 43) = 2.11, *p* = 0.153. PD patients tended to report more difficulties than their informants, while PSP patients reported fewer, but this group difference was not statistically reliable.

Overall, both PSP patients and their caregivers underreported executive dysfunction compared to objective impairment, indicating executive anosognosia. In contrast, PD patients and their informants tended to overreport difficulties, and there was no significant difference between patient and informant reports across groups.

#### Far-transfer discrepancy (ONA-CEF, AAPI-CP-r)

ANOVA revealed a significant group difference (Figure 1D; *F*(1, 36) = 8.20, *p* < 0.01, *η*^2^*ₚ* = 0.186). PSP patients underreported far-transfer difficulties relative to their objective deficits, unlike PD patients.

#### Far-transfer discrepancy (ONA-CEF, AAPI-CP-i)

Informant ratings showed a trend toward significance, *F*(1, 33) = 3.92, *p* = 0.056. Caregivers of PSP patients underreported their relatives’ impairment.

#### Far-transfer discrepancy (AAPI-CP-r, AAPI-CP-i)

A marginal group effect emerged for patient-informant differences, *F*(1, 37) = 3.18, *p* = 0.083, suggesting possible underreporting by PSP patients compared to their caregivers.

Overall, PSP patients consistently underreported cognitive and participative difficulties relative to objective performance and informant ratings.

Patients with PSP demonstrated significantly poorer objective executive functioning than patients with PD. PSP patients and their caregivers reported larger discrepancies between objective performance and self-reported executive difficulties, which is consistent with executive anosognosia. In contrast, PD patients and informants tended to overreport executive dysfunction. A similar pattern was seen for cognitive and participative difficulties. Although patient-informant discrepancies were not significant, the trends suggest that PSP patients may underreport difficulties slightly more than informants.

### Correlation analyses

As shown in Table 3, there is a clear dissociation between objective executive performance (z(ONA-CEF)) and subjective complaints (z(DEX-R), z(AAPI-CP)), with no significant correlations between them. Objective executive capacity does not predict perceived executive or participative difficulties. This suggests that ONA-CEF performance may not translate to everyday cognitive challenges.

**Table 3:** Correlations.

Variable		z(ONA-CEF)	z(DEX-R-r)	z(DEX-R-i)	z(AAPI-CP-r)	z(AAPI-CP-i)	z(AAPI-MA-r)	z(AAPI-MA-i)	z(BDI-FS)
1. z(ONA-CEF)	*n*	–							
	Pearson’s *r*	–							
	*p*-value	–							
	Spearman’s rho	–							
	*p*-value	–							
2. z(DEX-R-r)	*n*	41	–						
	Pearson’s *r*	**0.085**	–						
	*p*-value	0.598	–						
	Spearman’s rho	**0.148**	–						
	*p*-value	0.355	–						
3. z(DEX-R-i)	*n*	37	45	–					
	Pearson’s *r*	−0.104	0.416	–					
	*p*-value	0.539	0.004	–					
	Spearman’s rho	−0.138	0.368	–					
	*p*-value	0.415	0.013	–					
4. z(AAPI-CP-r)	*n*	38	49	42	–				
	Pearson’s *r*	−0.041	−0.733	−0.355	–				
	*p*-value	0.807	<0.001	0.021	–				
	Spearman’s rho	−0.083	−0.712	−0.282	–				
	*p*-value	0.618	<0.001	0.070	–				
5. z(AAPI-CP-i)	*n*	35	41	41	39	–			
	Pearson’s *r*	−0.006	−0.361	−0.820	0.388	–			
	*p*-value	0.972	0.020	<0.001	0.015	–			
	Spearman’s rho	0.079	−0.363	−0.845	0.469	–			
	*p*-value	0.652	0.020	<0.001	0.003	–			
6. z(AAPI-MA-r)	*n*	37	48	41	47	40	–		
	Pearson’s *r*	−0.110	−0.354	−0.124	0.542	0.334	–		
	*p*-value	0.517	0.013	0.439	<0.001	0.035	–		
	Spearman’s rho	−0.115	−0.393	−0.063	0.597	0.336	–		
	*p*-value	0.499	0.006	0.697	<0.001	0.034	–		
7. z(AAPI-MA-i)	*n*	35	41	41	39	43	40	–	
	Pearson’s *r*	−0.014	−0.240	−0.375	0.455	0.594	0.838	–	
	*p*-value	0.935	0.131	0.016	0.004	<0.001	<0.001	–	
	Spearman’s rho	0.003	−0.211	−0.393	0.480	0.623	0.783	–	
	*p*-value	0.988	0.185	0.011	0.002	<0.001	<0.001	–	
8. z(BDI-FS)	*n*	40	52	46	48	42	48	42	–
	Pearson’s *r*	**−0.057**	**0.608**	0.427	−0.654	−0.256	−0.260	−0.128	–
	*p*-value	0.728	<0.001	0.003	<0.001	0.102	0.075	0.419	–
	Spearman’s rho	**−0.019**	**0.532**	0.376	−0.561	−0.324	−0.391	−0.224	–
	*p*-value	0.906	<0.001	0.010	<0.001	0.036	0.006	0.154	–

*p < 0.05, **p < 0.01, ***p < 0.001. ONA-CEF: objective neuropsychological assessment-composite executive function; DEX-R: dysexecutive questionnaire revised; DEX-R-r: patient-reported DEX-R; DEX-R-i: informant-reported DEX-R; AAPI-CP: Aachen activity and participation index: cognition and participation; AAPI-CP-r: patient-reported AAPI-CP; AAPI-CP-i: informant-reported; AAPI-MA: Aachen activity and participation index: mobility and activity; AAPI-MA-r: patient-reported AAPI-MA; AAPI-MA-i: informant-reported AAPI-MA; BDI-FS: beck depression inventory–fast screen. The correlations highlighted in bold were subjected to a *z*-test for comparing dependent correlations (see text).

In contrast, depressive symptoms (z(BDI-FS)) were strongly correlated with subjective executive and broader cognitive-participative complaints. *Z*-tests confirmed that the correlation between DEX-R and BDI-FS was significantly stronger than the correlation between DEX-R and ONA-CEF (for Pearson correlations, z = −2.528, *p* < 0.01; for Spearman correlations, z = −1.841, *p* < 0.05).

These findings suggest that mood is related to subjective reports of executive and participative difficulties, while objective performance measures remain independent of depressive symptoms. This underscores the importance of distinguishing between objective executive assessments and mood-sensitive self-reports in clinical evaluations.

## Discussion

Our study demonstrates that patients with PSP exhibit significantly poorer executive functioning and pronounced executive anosognosia compared to patients with PD. PSP patients scored markedly lower on a reliability-optimized composite executive metric, yet neither they nor their caregivers reported greater executive dysfunction. By contrast, PD patients and their informants tended to overreport difficulties relative to objective performance. These findings reveal a dissociation between executive impairment and subjective awareness in PSP, with important implications for diagnosis, caregiver support, and outcome measurement.

PSP patients performed worse than PD patients on objective executive tasks, particularly verbal fluency. However, both PSP patients and caregivers consistently underreported deficits, evidenced by significant negative near-transfer and far-transfer discrepancy scores. In contrast, PD patients and informants often overestimated impairments. The magnitude and direction of discrepancies in PSP support the presence of executive anosognosia, likely reflecting frontal circuit pathology and impaired metacognitive insight (32). Notably, even informant-rated DEX-R scores underestimated dysfunction in PSP, possibly due to subtle behavioral manifestations or difficulty detecting executive failures in daily life.

Subjective complaints were more strongly correlated with depressive symptoms than with objective executive performance, indicating mood-related reporting bias. This aligns with prior findings in PD showing weak objective–subjective correspondence and the tendency of depression to inflate cognitive complaints (33). While the DEX-R demonstrates ecological validity in capturing perceived difficulties, its susceptibility to mood effects limits its construct validity as a standalone executive measure in populations with impaired insight.

Our composite measure identified verbal fluency as the most discriminative task, consistent with prior work showing that semantic and phonemic fluency differentiate PSP from PD with over 90% accuracy (34, 35). In contrast, indices from the WCST were less sensitive, with both groups performing poorly (36). Although the WCST is a well-established marker of general executive dysfunction, it lacks specificity for PSP versus PD, making it less suitable as a differential diagnostic tool. These findings underscore the need for psychometrically robust, multidimensional executive assessments that are sensitive to group differences.

### Implications for clinical assessment and research

Our results highlight the risks of relying solely on patient-reported outcome measures (PROMs) for assessing executive dysfunction, particularly in populations with anosognosia. Subjective questionnaires, even with informant versions, may underestimate impairment, while mood can inflate perceived deficits. In both clinical trials and routine care, uncritical use of PROMs risks underestimating executive dysfunction. Although regulatory guidance emphasizes psychometric validation, it often overlooks metacognitive deficits that impair accurate self-report (37). Thus, assessments should integrate objective, performance-based tasks (e.g., composite executive metrics), informant reports, and, where feasible, digital monitoring of everyday behavior to better evaluate functioning and treatment response (37, 38).

The discrepancy between performance-based and self-report measures reflects broader conceptual ambiguity in executive function research. Performance tasks tap discrete processes (e.g., set-shifting, inhibition), whereas self-reports assess broad, context-dependent behaviors (e.g., planning, persistence). Their weak correlation may suggest that core executive processes operate outside conscious awareness (39). Alternatively, instruments labeled “executive” may target fundamentally different constructs (40). Future research should examine extrinsic convergent validity—i.e., whether diverse measures predict shared real-world outcomes—to clarify method-specific versus common variance. Multimethod models incorporating tasks, self- and informant reports, and behavioral monitoring are needed to distinguish executive dysfunction from measurement artifacts.

### Limitations and future directions

Our cross-sectional design limits conclusions about the progression of anosognosia and executive dysfunction. Longitudinal studies are needed. The composite measure may overlook everyday aspects of executive function, and the modest sample limits generalizability. In the absence of healthy controls, objective performance and discrepancy measures cannot be interpreted against normative reference values. Without imaging or biomarkers, links to frontal pathology remain speculative. Informant reports may miss subtle dysfunction; digital tools could improve ecological validity. Moreover, performance on neuropsychological tests—particularly those assessing isolated cognitive processes—does not necessarily transfer to everyday functioning, especially in familiar and highly scaffolded environments. Consequently, concordant patient–informant ratings of everyday function may reflect preserved coping strategies or environmental support rather than intact or impaired awareness per se. Second, discrepancies (or the absence thereof) between objective test performance and subjective reports cannot unambiguously be interpreted as evidence for or against anosognosia. Stable self- and informant-reported functioning may coexist with subtle objective decline, and conversely, agreement between reports does not rule out limited ecological validity of the neuropsychological measures employed. Accordingly, the present findings should be interpreted with caution, and conclusions regarding awareness are necessarily relative rather than absolute. A further limitation concerns the interpretation of discrepancy scores based on sample-derived z-standardization. Although this approach was intended to characterize relative positioning within the study sample rather than absolute clinical impairment, combining sample-standardized objective performance with subjective ratings complicates the interpretation of constructs such as apparent sample-relative overreporting. These observed discrepancies may reflect distributional characteristics of the sample rather than true mismatches between perceived and actual functioning, particularly when normative benchmarks and guideline-based severity thresholds are not applied. Consequently, individuals who appear average within the sample may nonetheless show clinically relevant impairment relative to normative data, and subjective ratings that appear inflated in sample-internal comparisons may still be accurate. The absence of normative scoring therefore limits the clinical interpretability of these findings.

Future studies should use multimodal, longitudinal designs integrating cognitive testing, neuroimaging, repeated subjective ratings, and real-world monitoring. Psychometric validation of tools like the DEX-R and AAPI-CP is essential. Broader test batteries and larger cohorts can clarify domains prone to unawareness and identify key moderators. In addition, future work should clarify whether other atypical parkinsonian syndromes (e.g., multiple system atrophy) exhibit similar or distinct patterns of anosognosia.

## Conclusion

Our findings demonstrate that patients with PSP exhibit executive anosognosia, underestimating their deficits despite clear objective impairment. In contrast, PD patients and their informants tend to overreport dysfunction relative to performance. Subjective ratings were more strongly associated with mood than with executive capacity, limiting their utility as standalone measures. These results underscore the need to combine patient-reported outcomes with objective, performance-based assessments—and, where possible, real-world behavioral data—to capture executive dysfunction accurately.

## Data Availability

The raw data supporting the conclusions of this article will be made available by the authors, without undue reservation.

## Footnotes

1
https://jasp-stats.org/

2
https://www.psychometrica.de/
