# Supplementary material for: Executive anosognosia in progressive supranuclear palsy versus Parkinson’s disease
Source: Front Neurol. 2026 Feb 2;17:1744979. doi: 10.3389/fneur.2026.1744979 (PMC12907162; doi:10.3389/fneur.2026.1744979)
Supplement: Supplementary file 1 [file Table_1.docx]

***Supplementary Material***

**1 Supplementary Data**

1. The extended CERAD NAB Plus.

The core CERAD NAB includes Animal Fluency, which assesses semantic verbal fluency by counting the number of animals named within one minute. The abbreviated Boston Naming Test evaluates word retrieval by having individuals correctly name 15 objects. The Word List Learning task measures immediate verbal memory by having individuals recall a list of ten words over three trials, for a maximum score of thirty. After a delay period, the Word List Recall task assesses the ability to recall the same 10 words after a delay, with a maximum score of 10. The Word List Intrusions task tallies the number of incorrect words recalled during the learning and recall phases. Word List Savings is calculated by dividing the number of words recalled after the delay by the number recalled in the third learning trial and multiplying the result by 100. This reflects retention efficiency. The Word List Discriminability task evaluates recognition memory by presenting 20 words (10 targets and 10 distractors), and the score is derived from correct identifications adjusted for false positives.

The Constructional Praxis test assesses visuospatial skills by having individuals copy four geometric figures. The maximum score is 11. After a delay, the Constructional Praxis Recall test assesses the ability to reproduce these figures from memory. The maximum score is also 11. Constructional Praxis Savings is determined by dividing the recall score by the initial copy score and multiplying by 100. This indicates visual memory retention.

The extended CERAD NAB Plus includes Letter Fluency, in which individuals generate as many words as possible beginning with the letter "S" in one minute to assess phonemic verbal fluency. The Trail Making Test A measures processing speed by timing the connection of numbered dots within a maximum time of 180 seconds. The Trail Making Test B assesses executive function by requiring alternation between numbers and letters in sequence within a maximum time of 300 seconds.

These 13 scores collectively provide a multidimensional profile of cognitive abilities, facilitating the detection and monitoring of impairments associated with neurodegenerative conditions. The extended CERAD NAB Plus has been standardized for use in German-speaking countries. Normative data were collected from 1,100 healthy individuals, aged 49 to 92, for the core tests and from 604 individuals, aged 50 to 88, for the additional tests. Standardization allows for the calculation of z-scores adjusted for demographic variables (age, education, and gender), which facilitates a precise assessment of cognitive performance. These z-scores can be obtained through an online platform, which is provided by the Memory Clinic at the University Hospital Basel and is available at www.memoryclinic.ch.

1. The Modified Wisconsin Card Sorting Test [(1)](https://paperpile.com/c/QHP0pn/xNOI)

This streamlined, paper-and-pencil version of the original Wisconsin Card Sorting Test is commercially available (PAR). It includes four fixed stimulus cards featuring a red triangle, two green stars, three yellow crosses, and four blue circles. Participants are asked to sort 48 response cards by matching each to one of the stimulus cards based on color, shape, or number. They do not know the correct sorting rule in advance. After each card placement, the examiner provides immediate feedback ("correct" or "incorrect"), and the sorting rule changes after six consecutive correct responses. This requires participants to adapt to new categorization criteria.

The M-WCST was standardized using a normative sample of 323 healthy individuals ranging from 18 to over 85 years of age (with a mean age of around 55 years), who were recruited via random sampling. These normative data allow individual performance to be adjusted for age, education, and gender using z-scores. This makes the M-WCST a reliable and valid tool for assessing executive dysfunction in clinical and research settings [(2,3)](https://paperpile.com/c/QHP0pn/abPxj+ux14g).

1. Pearson and Spearman partial correlations conditioned on age

Using partial correlations conditioned on age revealed very similar results (see Table S1). Partial correlations between *z*(ONA-CEF) and patient- and informant-reported *z*(DEX-R) and *z*(AAPI-CP) remained negligible, while partial correlations between z(BDI-FS) and *z*(DEX-R) and *z*(AAPI-CP) remained substantial.

A *z*-test for comparing dependent partial correlations revealed that the association between DEX-R-r and ONA-CEF (*r* = -0.002) was significantly lower than that between DEX-R-r and BDI-FS (*r* = 0.645), *z* = –3.115, *p* < .01. A similar result emerged when using Spearman’s rho: the partial correlation between DEX-R-r and ONA-CEF (*ρ* = 0.007) was again significantly lower than that with BDI-FS (*ρ* = 0.547), *z* = –2.509, *p* < .01.

*Synthesis*

Thus, after controlling for age, partial correlations revealed that the association between z(ONA-CEF) and patient-reported executive ratings was weaker than the association between depressive mood and these subjective reports, which remained substantial.

Reference

1. [Schretlen DJ, van der Hulst E-J, Pearlson GD, Gordon B. A neuropsychological study of personality: trait openness in relation to intelligence, fluency, and executive functioning. *J Clin Exp Neuropsychol* (2010) 32:1068–1073.](http://paperpile.com/b/QHP0pn/xNOI)

2. [Kopp B, Lange F, Steinke A. The Reliability of the Wisconsin Card Sorting Test in Clinical Practice. *Assessment* (2021) 28:248–263.](http://paperpile.com/b/QHP0pn/abPxj)

3. [Faber D, Grosse GM, Klietz M, Petri S, Schwenkenbecher P, Sühs K-W, Kopp B. Towards the Validation of Executive Functioning Assessments: A Clinical Study. *J Clin Med* (2022) 11: doi:](http://paperpile.com/b/QHP0pn/ux14g) [10.3390/jcm11237138](http://dx.doi.org/10.3390/jcm11237138)

**2 Supplementary table**

| Partial Correlation Table | | | | | | | | | | | | | | | | | | | |
| --- | --- | --- | --- | --- | --- | --- | --- | --- | --- | --- | --- | --- | --- | --- | --- | --- | --- | --- | --- |
| Variable | |  | | z(ONA-CEF) | | z(DEX-R-r) | | z(DEX-R-i) | | z(AAPI-CP-r) | | z(AAPI-CP-i) | | z(AAPI-MA-r) | | z(AAPI-MA-i) | | z(BDI-FS) | |
| 1. z(ONA-CEF) |  | n |  | — |  |  |  |  |  |  |  |  |  |  |  |  |  |  |  |
|  |  | Pearson's r |  | — |  |  |  |  |  |  |  |  |  |  |  |  |  |  |  |
|  |  | p-value |  | — |  |  |  |  |  |  |  |  |  |  |  |  |  |  |  |
|  |  | Spearman's rho |  | — |  |  |  |  |  |  |  |  |  |  |  |  |  |  |  |
|  |  | p-value |  | — |  |  |  |  |  |  |  |  |  |  |  |  |  |  |  |
| 2. z(DEX-R-r) |  | n |  | 41 |  | — |  |  |  |  |  |  |  |  |  |  |  |  |  |
|  |  | Pearson's r |  | **-0.002** |  | — |  |  |  |  |  |  |  |  |  |  |  |  |  |
|  |  | p-value |  | 0.988 |  | — |  |  |  |  |  |  |  |  |  |  |  |  |  |
|  |  | Spearman's rho |  | **0.070** |  | — |  |  |  |  |  |  |  |  |  |  |  |  |  |
|  |  | p-value |  | 0.669 |  | — |  |  |  |  |  |  |  |  |  |  |  |  |  |
| 3. z(DEX-R-i) |  | n |  | 37 |  | 45 |  | — |  |  |  |  |  |  |  |  |  |  |  |
|  |  | Pearson's r |  | -0.143 |  | 0.423 | ** | — |  |  |  |  |  |  |  |  |  |  |  |
|  |  | p-value |  | 0.404 |  | 0.004 |  | — |  |  |  |  |  |  |  |  |  |  |  |
|  |  | Spearman's rho |  | -0.168 |  | 0.371 | * | — |  |  |  |  |  |  |  |  |  |  |  |
|  |  | p-value |  | 0.326 |  | 0.013 |  | — |  |  |  |  |  |  |  |  |  |  |  |
| 4. z(AAPI-CP-r) |  | n |  | 38 |  | 49 |  | 42 |  | — |  |  |  |  |  |  |  |  |  |
|  |  | Pearson's r |  | 0.062 |  | -0.730 | *** | -0.369 | * | — |  |  |  |  |  |  |  |  |  |
|  |  | p-value |  | 0.714 |  | < .001 |  | 0.018 |  | — |  |  |  |  |  |  |  |  |  |
|  |  | Spearman's rho |  | -0.006 |  | -0.709 | *** | -0.288 |  | — |  |  |  |  |  |  |  |  |  |
|  |  | p-value |  | 0.970 |  | < .001 |  | 0.068 |  | — |  |  |  |  |  |  |  |  |  |
| 5. z(AAPI-CP-i) |  | n |  | 35 |  | 41 |  | 41 |  | 39 |  | — |  |  |  |  |  |  |  |
|  |  | Pearson's r |  | 0.044 |  | -0.356 | * | -0.830 | *** | 0.384 | * | — |  |  |  |  |  |  |  |
|  |  | p-value |  | 0.806 |  | 0.024 |  | < .001 |  | 0.017 |  | — |  |  |  |  |  |  |  |
|  |  | Spearman's rho |  | 0.133 |  | -0.359 | * | -0.856 | *** | 0.468 | ** | — |  |  |  |  |  |  |  |
|  |  | p-value |  | 0.453 |  | 0.023 |  | < .001 |  | 0.003 |  | — |  |  |  |  |  |  |  |
| 6. z(AAPI-MA-r) |  | n |  | 37 |  | 48 |  | 41 |  | 47 |  | 40 |  | — |  |  |  |  |  |
|  |  | Pearson's r |  | 0.044 |  | -0.299 | * | -0.165 |  | 0.543 | *** | 0.338 | * | — |  |  |  |  |  |
|  |  | p-value |  | 0.801 |  | 0.041 |  | 0.308 |  | < .001 |  | 0.036 |  | — |  |  |  |  |  |
|  |  | Spearman's rho |  | 0.046 |  | -0.327 | * | -0.085 |  | 0.610 | *** | 0.349 | * | — |  |  |  |  |  |
|  |  | p-value |  | 0.789 |  | 0.025 |  | 0.604 |  | < .001 |  | 0.029 |  | — |  |  |  |  |  |
| 7. z(AAPI-MA-i) |  | n |  | 35 |  | 41 |  | 41 |  | 39 |  | 43 |  | 40 |  | — |  |  |  |
|  |  | Pearson's r |  | 0.114 |  | -0.205 |  | -0.441 | ** | 0.434 | ** | 0.600 | *** | 0.803 | *** | — |  |  |  |
|  |  | p-value |  | 0.520 |  | 0.205 |  | 0.004 |  | 0.007 |  | < .001 |  | < .001 |  | — |  |  |  |
|  |  | Spearman's rho |  | 0.107 |  | -0.179 |  | -0.453 | ** | 0.480 | ** | 0.633 | *** | 0.736 | *** | — |  |  |  |
|  |  | p-value |  | 0.549 |  | 0.270 |  | 0.003 |  | 0.002 |  | < .001 |  | < .001 |  | — |  |  |  |
| 8. z(BDI-FS) |  | n |  | 40 |  | 52 |  | 46 |  | 48 |  | 42 |  | 48 |  | 42 |  | — |  |
|  |  | Pearson's r |  | **-0.077** |  | **0.645** | *** | 0.426 | ** | -0.692 | *** | -0.278 |  | -0.371 | * | -0.224 |  | — |  |
|  |  | p-value |  | 0.641 |  | < .001 |  | 0.004 |  | < .001 |  | 0.079 |  | 0.010 |  | 0.160 |  | — |  |
|  |  | Spearman's rho |  | **-0.045** |  | **0.547** | *** | 0.375 | * | -0.575 | *** | -0.336 | * | -0.469 | *** | -0.294 |  | — |  |
|  |  | p-value |  | 0.785 |  | < .001 |  | 0.011 |  | < .001 |  | 0.032 |  | < .001 |  | 0.062 |  | — |  |
|  | | | | | | | | | | | | | | | | | | | |
| * p < .05, ** p < .01, *** p < .001 | | | | | | | | | | | | | | | | | | | |
| *Note.*  Standard error of effect size (Fisher's z) is currently unavailable for non-parametric partial correlations. | | | | | | | | | | | | | | | | | | | |
| *Note.*  Conditioned on variables: Age. | | | | | | | | | | | | | | | | | | | |

Abbreviations: ONA-CEF: Objective Neuropsychological Assessment-Composite Executive Function; DEX-R: Dysexecutive Questionnaire Revised; DEX-R-r: patient-reported DEX-R; DEX-R-i: informant-reported DEX-R; AAPI-CP: Aachen Activity and Participation Index: Cognition and Participation; AAPI-CP-r: patient-reported AAPI-CP; AAPI-CP-i: informant-reported; AAPI-MA: Aachen Activity and Participation Index: Mobility and Activity; AAPI-MA-r: patient-reported AAPI-MA; AAPI-MA-i: informant-reported AAPI-MA;BDI-FS: Beck Depression Inventory–Fast Screen. The correlations highlighted in bold were subjected to a *z*-test for comparing dependent partial correlations (see text).
